# Utilizing murine dendritic cell line DC2.4 to evaluate the immunogenicity of subunit vaccines *in vitro*

**DOI:** 10.3389/fimmu.2024.1298721

**Published:** 2024-02-26

**Authors:** Lantian Lu, Wei Yang Kong, Jiahui Zhang, Farrhana Firdaus, James W. Wells, Rachel J. Stephenson, Istvan Toth, Mariusz Skwarczynski, Jazmina L. Gonzalez Cruz

**Affiliations:** ^1^ School of Chemistry and Molecular Biosciences, The University of Queensland, St. Lucia, QLD, Australia; ^2^ Faculty of Medicine, Frazer Institute, The University of Queensland, Woolloongabba, QLD, Australia; ^3^ Institute of Molecular Bioscience, The University of Queensland, St. Lucia, QLD, Australia; ^4^ School of Pharmacy, The University of Queensland, Woolloongabba, QLD, Australia

**Keywords:** DC2.4, dendritic cell uptake, dendritic cell maturation, *in vitro* assay, subunit vaccine

## Abstract

Subunit vaccines hold substantial promise in controlling infectious diseases, due to their superior safety profile, specific immunogenicity, simplified manufacturing processes, and well-defined chemical compositions. One of the most important end-targets of vaccines is a subset of lymphocytes originating from the thymus, known as T cells, which possess the ability to mount an antigen-specific immune response. Furthermore, vaccines confer long-term immunity through the generation of memory T cell pools. Dendritic cells are essential for the activation of T cells and the induction of adaptive immunity, making them key for the *in vitro* evaluation of vaccine efficacy. Upon internalization by dendritic cells, vaccine-bearing antigens are processed, and suitable fragments are presented to T cells by major histocompatibility complex (MHC) molecules. In addition, DCs can secrete various cytokines to crosstalk with T cells to coordinate subsequent immune responses. Here, we generated an *in vitro* model using the immortalized murine dendritic cell line, DC2.4, to recapitulate the process of antigen uptake and DC maturation, measured as the elevation of CD40, MHC-II, CD80 and CD86 on the cell surface. The levels of key DC cytokines, tumor necrosis alpha (TNF-α) and interleukin-10 (IL-10) were measured to better define DC activation. This information served as a cost-effective and rapid proxy for assessing the antigen presentation efficacy of various vaccine formulations, demonstrating a strong correlation with previously published *in vivo* study outcomes. Hence, our assay enables the selection of the lead vaccine candidates based on DC activation capacity prior to *in vivo* animal studies.

## 1 Introduction

Vaccines are critical tools for providing immunity against various pathogens and cancer malignancies (1). In recent years, peptide-based subunit vaccines have gained increasing research interest due to their superior safety profile and specific immunogenicity. However, these vaccines often exhibit lower immunogenicity when compared to conventional whole-pathogen vaccines, necessitating the use of immunopotentiators, such as adjuvants, to enhance the magnitude of the immune response (2). Adjuvants play pivotal roles in cell signaling, stimulating the innate immunity in the preamble to a robust antigen-specific adaptive immunity. Without the presence of an adjuvant, an antigen may induce immune tolerance rather than activating the immune system (3–5). Therefore, it is essential to co-deliver an adjuvant with an antigen to induce a desired immune response.

The assessment of vaccine efficacy is often carried out using *in vivo* models. However, these studies not only require specialized facilities and personnel but also necessitate the use of a significant number of experimental animals and/or human subjects. Consequently, there is a critical imperative to reduce animal usage as much as feasibly possible, to address ethical concerns and to adhere to the 3Rs (Replacement, Reduction, and Refinement) principles governing animal research (6). One strategy to address this issue is to perform *in vitro* evaluations before proceeding to *in vivo* assessments. This helps to reduce the number of experimental animals used by eliminating formulations that fail to demonstrate promise during the *in vitro* evaluation stage, thus precluding their advancement to further *in vivo* testing (7). However, this depends on a proven correlation between the results of *in vitro* and *in vivo* evaluations. Moreover, conducting *in vitro* studies is also important to elucidate the mechanisms that underpin vaccine-mediated protection or failure.

The generation and expansion of antigen-specific T cells is one of the end goals of all vaccines. There are two main T cell linages: CD4^+^ T cells and CD8^+^ T cells. CD4^+^ T cells, also known as helper T cells, play a crucial role in activating other immune cells, including B cells which are responsible for the initiation of the humoral response, and CD8^+^ T cells which are central to the adaptive immunity cytotoxic response. To activate T cells, professional antigen-presenting cells (APCs), such as dendritic cells (DCs), present fragmented exogenous or endogenous peptide antigens to naïve T cells through MHC molecules. While various DC sources have been used to evaluate the immunogenicity of vaccines or adjuvants in different assays, limited DC-based assays employing a single cell line have been reported for evaluating the efficacy of peptide vaccines (8, 9).

The DC2.4 cell line, derived from C57BL/6 mice, is an immortalized murine DC line generated through retrovirus transduction of oncogenes *myc* and *raf* (10). These cells express DC-specific markers, including MHC class I (MHC-I), MHC-II, B7-1 (CD80), B7-2 (CD86), as well as CD32 and CD54, and possess the ability to present antigens on both MHC-I and -II molecules (11). These properties have made the DC2.4 cell line particularly valuable for assessing the immunogenicity of vaccines *in vitro*. Herein, we present a detailed step-by-step protocol for the DC uptake and maturation assays utilizing the DC2.4 cell line to facilitate the evaluation of the immunogenicity of peptide-based vaccines. This protocol provides robust and scalable assays with high-throughput potential to identify peptide vaccine candidates with the highest prospects of eliciting humoral/cellular responses *in vivo*.

## 2 Materials and equipment

### 2.1 Cells and media

Immortalized DC2.4 cell line (SCC142) was purchased from Merck (Rahway, United States) and cultured in RPMI-1640 medium supplemented with 10% v/v fetal bovine serum (FBS) (Thermo Fisher Scientific, Waltham, United States), 2.5% v/v HEPES (1M) buffer (Thermo Fisher Scientific), 1% v/v L-glutamine (Thermo Fisher Scientific), 1% v/v MEM Non-essential Amino Acids Solution (100X) (NEAA), 1% v/v Penicillin-Streptomycin-Glutamine (100X) (PSG), and 0.00054% v/v 2-mercaptoethanol (Thermo Fisher Scientific, Waltham, United States). Cells for cryopreservation were resuspended in freezing media containing 90% v/v FBS and 10% dimethyl sulfoxide (DMSO; Merck, Rahway, United States). Trypsin-EDTA solution 1X (Merck, Rahway, United States) was used for cell dissociation.

### 2.2 Antibodies, dyes and beads

The list of antibodies used for flow cytometry is summarized in **Table 1**. LIVE/DEAD™ Fixable Aqua Dead Cell Stain Kit was purchased from BioLegend (San Diego, United States). Anti-rat and Anti-hamster Igκ/Negative Control Compensation Beads were purchased from BD Biosciences (Franklin Lakes, United States). Fluorescein isothiocyanate (FITC)-dextran (4 kDa, 25 mg/ml x 5 ml) was purchased from Chondrex (Woodinville, United States).

**Table 1:** The list of antibodies used in this study.

Antibody	Cat number	Manufacturer
Alexa Fluor^®^ 488 anti-mouse langerin	53-2073-80	eBioscience
Alexa Fluor^®^ 700 anti-mouse CD45R/B200	103231	Biolegend
APC anti-mouse H-2Kb	116619	Biolegend
APC-Cy7 anti-mouse I-A/I-E	107628	Biolegend
Brilliant Violet 421™ anti-mouse CD80	104725	Biolegend
Brilliant Violet 421™ anti-mouse CD86	105031	Biolegend
Brilliant Violet 605™ anti-mouse CD8a	100744	Biolegend
Brilliant Violet 650™ anti-mouse CD11b	101239	Biolegend
Brilliant Violet 711™ anti-mouse F4/80	123147	Biolegend
Brilliant Violet 785™ anti-mouse CD86	105043	Biolegend
Brilliant Violet 785™ anti-mouse TNF-α	506341	Biolegend
FITC anti-mouse CD80	104705	Biolegend
PE anti-mouse CD40	124609	Biolegend
PE anti-mouse IL-10	505008	Biolegend
PE/Cyanine7 anti-mouse CD11c	117317	Biolegend
PerCP/Cyanine5.5 anti-mouse CD317 (BST2, PDCA-1)	127021	Biolegend
TruStain FcX™ (anti-mouse CD16/32)	101320	Biolegend

### 2.3 Flasks, plates, and tubes

T75 flasks were purchased from Thermo Fisher Scientific (Brisbane, Australia). Corning^®^ 50 mL centrifuge tubes, Corning^®^ 2 mL Internal Threaded Polypropylene Cryogenic Vials (self-standing with round bottom), Corning^®^ Costar^®^ TC-treated Multiple Well Plates, Greiner 96-well polypropylene V-bottom plates, and 1.5 mL microcentrifuge tubes were purchased from Merck (Rahway, United States).

### 2.4 Other reagents

Gibco 1X phosphate buffered saline (1X PBS) pH 7.4, trypan blue solution (0.4%) and lipopolysaccharide solution (500X) were purchased from Thermo Fisher Scientific (Brisbane, Australia). Paraformaldehyde aqueous solution (16% PFA) was purchased from Emgrid Australia (Gulfview Heights, Australia) and diluted to 4% using 1X PBS. Albumin from chicken egg white (OVA) was purchased from Merck (Rahway, United States). Recombinant murine interferon-gamma (IFN-γ) was purchased from Peprotech (Cranbury, United States). Pam_2_CSK_4_ (trifluoroacetate salt) was purchased from Sapphire Bioscience Pty Limited (Redfern, Australia).

### 2.5 Equipment

- BD LSRFortessa™ X-20 Cell Analyzer (BD Biosciences, Franklin Lakes, United States).
- TC20 Automated Cell Counter (Bio-Rad, Hercules, United States).
- Mr. Frosty™ Freezing Container (Thermo Fisher Scientific, Waltham, United States).
- CO_2_ Incubator (Panasonic, Kadoma, Japan).
- CKX31 Inverted Microscope (Olympus Life Science, Waltham, United States).
- ESCO Biological Safety Cabinet (ESCO Lifesciences, Upper Changi, Singapore).

### 2.6 Software

- FlowJo (version 10.8.1, BD Biosciences, Franklin Lakes, USA).
- GraphPad Prism (version 9.0, GraphPad, San Diego, USA).

## 3 Methods

### 3.1 Synthesis and purification of vaccine candidates

All peptides used for maturation assays were synthesized using butyloxycarbonyl (Boc) solid-phase peptide synthesis (12, 13). Boc-protected L-amino acids were assembled on p-methyl-benzhydryl-amine hydrochloride (pMBHA·HCl) resin at 0.2 mmol scale, following the previously reported method (12, 13).

In brief, the resin was weighed and swelled in *N,N*-dimethylformamide (DMF) and *N,N*-diisopropylethylamine (DIPEA) (6.2 eq.) overnight. The coupling cycle for Boc synthesis included deprotection of the Boc group (1 min treatment with TFA, twice at ambient conditions), DMF wash, addition of activated amino acids (0.84 mmol/g, 4.2 eq.) by 0.5 M hexafluorophosphate azabenzotriazole tetramethyl uranium (HATU) (1.6 mL, 4.0 eq.) and DIPEA (0.26 mL, 6.2 eq.), and coupling (10 min and 20 min at RT, respectively). After coupling, the liquid content was aspirated, and the abovementioned steps were repeated until the desired peptide sequence was achieved. For Boc-Gln (Xan)-OH, dichloromethane (DCM) was used to wash between the two trifluoroacetic acid (TFA) deprotection steps to avoid glutamine cyclization. Acetylation was performed after the last amino acid was coupled using acetylation solution (5% DIPEA and 5% acetic anhydride in DMF). The formyl group from tryptophan was removed using 20% piperidine in DMF solution (5 min and 10 min, respectively). The resin was then washed with DMF (3X), followed by DCM (3X), and methanol (1X) before transferring the resin-peptide to a desiccator overnight.

The peptides were cleaved from the resin using anhydrous hydrogen fluoride (HF) with *p*-cresol and/or *p*-thiocresol as scavengers (14). Upon evaporation of HF, the cleaved peptides were washed with cold diethyl ether and/or mixture of diethyl ether and *n*-hexane (1:1). The precipitated compounds were dissolved in mixture of solvent A (100% Milli-Q water containing 0.1% TFA) and solvent B (90% acetonitrile and 10% Milli-Q water containing 0.1% TFA) depending on the hydrophobicity of the peptide. After filtration, the peptide was purified using a Shimadzu preparative reversed-phase HPLC (RP-HPLC; Kyoto, Japan) instrument (LC-20AP x 2, CBM-20A, SPD-20A, FRC-10A) with a 20.0 mL/min flow rate on a C18 (218TP1022; 10 μm, 22 × 250 mm) or C4 (214TP1022; 10 μm, 22 × 250 mm) column depending on the hydrophobicity of the compound. Once purified, the purity of all peptides was determined using an analytical RP-HPLC on a C18 (218TP54; 5 μm,4.6 × 250 mm) or C4 (214TP54; 5 μm, 4.6 × 250 mm) Vydac column, with a 0 – 100% gradient of solvent B for 40 min at 214 nm. ESI-MS was performed on a LCMS-2020 Shimadzu (Kyoto, Japan) instrument (DGU-20A3, LC-20Ad x 2, SIL-20AHT, STO-20A) and Analyst 1.4 software (Applied Biosystems/MDS Sciex, Toronto, Canada) (Perkin-Elmer-Sciex API3000) to validate the molecular weight of the compound.

PADRE-J8 (AFKVAAWTLKAAA-QAEDKVKQSREAKKQVEKALKQLEDKVQ). Yield: 30%. Molecular weight: 4653.42 g/mol. ESI-MS [M + 3H]^3+^ m/z 1552.7 (calc. 1552.1), ESI-MS [M + 4H]^4+^ m/z 1164.5 (calc. 1164.4), [M + 5H]^5+^ m/z 932.1 (calc. 931.7), [M + 6H]^6+^ m/z 776.8 (calc. 776.6), [M + 7H]^7+^ m/z 666.0 (calc. 665.8). t_R_ = 24.5 min (0 to 100% solvent B; C18 column); purity ≥ 99%.

L_15_-PADRE-J8 (LLLLLLLLLLLLLLL-AFKVAAWTLKAAA-QAEDKVKQSREAKKQVEKALKQLEDKVQ). Yield: 3%. Molecular weight: 6350.82 g/mol. ESI-MS [M + 4H]^4+^ m/z 1588.3 (calc. 1588.7), [M + 5H]^5+^ m/z 1272.0 (calc. 1271.2), [M + 6H]^6+^ m/z 1060.3 (calc. 1059.5), [M + 7H]^7+^ m/z 908.0 (calc. 908.3), [M + 8H]^8+^ m/z 794 (calc. 794.9), [M + 9H]^9+^ m/z 705.5 (calc. 706.6). t_R_ = 39.0 min (0 to 100% solvent B; C4 column); purity ≥ 99%.

J8-K(V_10_)-PADRE (QAEDKVKQSREAKKQVEKALKQLEDKVQ-K(VVVVVVVVVV)-AFKVAAWTLKAAA). Yield: 23%. Molecular Weight: 5814.96. ESI-MS [M + 3H]^3+^ m/z 1938.6 (calc. 1939.3), [M + 4H]^4+^ m/z 1455.0 (calc. 1454.7), [M + 5H]^5+^ m/z 1164.0 (calc. 1164.0), [M + 6H]^6+^ m/z 969.9 (calc. 970.1), [M + 7H]^7+^ m/z 831.5 (calc. 831.7), [M + 8H]^8+^ m/z 728.0 (calc. 727.9). t_R_ = 24.1 min (0 to 100% solvent B; C4 column); purity ≥ 99%.

J8-K(F_10_)-PADRE (QAEDKVKQSREAKKQVEKALKQLEDKVQ-K(FFFFFFFFFF)- AFKVAAWTLKAAA). Yield: 28%. Molecular weight: 6295.40. ESI-MS [M + 4H]^4+^ m/z 1574.7 (calc. 1574.9), [M + 5H]^5+^ m/z 1260.0 (calc. 1260.0), [M + 6H]^6+^ m/z 1050.3 (calc. 1050.2), [M + 7H]^7+^ m/z 900.4 (calc. 900.3), [M + 8H]^8+^ m/z 788.0 (calc. 787.9), [M + 9H]^9+^ m/z 700.7 (calc. 700.5). t_R_ = 24.5 min (0 to 100% solvent B; C4 column); purity ≥ 99%.

J8-K(L_10_)-PADRE (QAEDKVKQSREAKKQVEKALKQLEDKVQ-K(LLLLLLLLLL)- AFKVAAWTLKAAA). Yield: 28%. Molecular weight: 5955.23. ESI-MS [M + 3H]^3+^ m/z 1985.2 (calc. 1986.1), [M + 4H]^4+^ m/z 1489.6 (calc. 1489.8), [M + 5H]^5+^ m/z 1192.0 (calc. 1192.0), [M + 6H]^6+^ m/z 993.5 (calc. 993.5), [M + 7H]7^+^ m/z 851.8 (calc. 851.7), [M + 8H]^8+^ m/z 745.4 (calc. 745.4). t_R_ = 25.6 min (0 to 100% solvent B; C4 column); purity ≥ 99%.

J8-K(L_15_)-PADRE (QAEDKVKQSREAKKQVEKALKQLEDKVQ-K(LLLLLLLLLLLLLLL)- AFKVAAWTLKAAA). Yield: 26%. Molecular weight: 6521.03. ESI-MS [M + 4H]^4+^ m/z 1631.8 (calc. 1631.3), [M + 5H]^5+^ m/z 1305.6 (calc. 1305.2), [M + 6H]^6+^ m/z 1088.0 (calc. 1087.8), [M + 7H]^7+^ m/z 932.9 (calc. 932.6), [M + 8H]^8+^ m/z 816.3 (calc. 816.1), [M + 9H]^9+^ m/z 725.6 (calc. 725.6). t_R_ = 30.9 min (0 to 100% solvent B; C4 column); purity ≥ 99%.

### 3.2 DC2.4 cells culturing protocol

**3.2.1** Prepare culture media by supplementing RPMI-1640 media with the following components: 10% v/v FBS, 2.5% v/v HEPES buffer, 1% v/v L-glutamine, 1% v/v NEAA, 1% v/v PSG, and 0.00054% v/v 2-mercaptomethanol, as described in **Section 2.1**.

**3.2.2** Thaw cryopreserved DC2.4 cells by placing the cryotube in a 37 °C water bath. Swirl the tube gently until only a small piece of ice remains. Add the cells dropwise using a serological pipette to 20 mL of pre-warmed (37 °C) media. Spin down the cells using a benchtop centrifuge at RT at 1,200 rpm (288 rcf) for 10 min and resuspend in 10 mL of media.

**3.2.3** Load 10 μL of the cell suspension and 10 μL of 0.4% trypan blue solution into a TC20™ cell counting slide and use a TC20 Automated Cell Counter to determine cell density. Seed 1x – 3x 10^6^ cells in a T75 flask for passaging and top up to 30 mL with culturing media. Transfer the flask to an incubator supplemented with 5% CO_2_ at 37 °C for cell culturing.

**3.2.4** Refresh the culture media every 24 hrs after cell seeding. Harvest the cells when the confluency reaches 60 – 80% (~2 – 4 days post-seeding).

**3.2.5** Remove the media using a serological pipette and add 5 mL of trypsin-EDTA 1X. Gently shake the flask at RT or incubate at 37 °C for 5 min until more than 80% of the cells can be seen detached under the microscope. Neutralize trypsin by adding double the volume (10 mL) of media and pipette up and down to help remove undetached cells. Collect the cells in a 50 mL Falcon tube and spin down at 1,200 rpm (288 rcf) at RT for 10 min. Remove the supernatant and suspend the pellet with 20 mL of media. Measure the cell density as described in **Step 3.2.3**.

**3.2.6** Seed the cells onto a 48-well plate at the required densities for uptake or maturation studies, as described in **Section 3.4** and **3.5**. Either spin the remaining cells down and resuspend in freezing media containing 90% FBS and 10% DMSO at 1 – 3 million cells/mL for cryopreservation, or passage the cells as described in **Step 3.2.3**.

### 3.3 DC characterization

**3.3.1** Prepare 2 x 10^5^ cells per sample for flow cytometry phenotyping of DC2.4 cells. Prepare whole panel-stained samples, as well as unstained, live/dead, and fluorescent minus one (FMO) controls as detailed in the following steps. For the live/dead control, add 50 µL of 80% ethanol and incubate for 5 min at RT.

**3.3.2** Wash the cells with 150 µL of PBS and spin down at 1,700 rpm (271 rcf) using a microcentrifuge for 5 min. Resuspend the cells in 100 µL of Zombie Aqua™ Live/Dead solution (1:200 diluted in 1X PBS) on ice in the dark for 10 min.

**3.3.3** Wash the cells once with PBS and resuspend the pellet in 100 µL of TruStain FcX™ solution (1:200 diluted in 1X PBS). Incubate on ice for 15 min, then spin down at 1,700 rpm (271 rcf) for 5 min.

**3.3.4** Prepare a cocktail containing all the antibodies listed in **Table 2** using FACs buffer. Add 100 µL to the cells, and resuspend the cells, label the sample as fully stained samples. For FMO controls, prepare 100 µL of antibody master mix excluding one of the antibodies in **Table 2**. Prepare a total of five FMOs, including CD11c-PE/Cy7 FMO, CD40-PE FMO, CD80-BV421 FMO, CD86-BV785 FMO, and MHC-I-APC FMO. Stain these samples mentioned in **Step 3.3.4** for 25 min on ice in the dark, followed by washing with 200 µL of 1X PBS to remove excess antibodies. Centrifuge the samples at 1,700 rpm (271 rcf) for 5 min and discard the supernatant.

**Table 2:** Antibody panel for DC2.4 characterization by flow cytometry.

Antibody	Dilution
MHC-II-APC/Cy7	1:800
B220-AF700	1:600
CD11b-BV650	1:400
CD40-PE	1:400
MHC-I-APC	1:400
CD317-PerCP/Cy5.5	1:200
CD8α-BV605	1:200
CD80-BV421	1:200
CD86-BV785	1:200
F4/80-BV711	1:200

**3.3.5** Wash the cells once with PBS and resuspend them in 100 µL of 4% PFA (diluted with 1X PBS) for 15 min at RT to fix the cells.

**3.3.6** Wash the fixed cells with PBS once, and resuspend them in 200 µL of PBS, then transfer them to a FACS tube for flow cytometry analysis.

**3.3.7** Before flow analysis, add a small aliquot of fixed untreated live cells to the live/dead control.

### 3.4 DC uptake protocol

**3.4.1** Prepare a 48-well plate and seed 9x10^4^ cells in each well with 1.0 mL of cell culturing media.

**3.4.2** Incubate the plate in an incubator supplemented with 5% CO_2_ at 37°C overnight (~18 hr). Afterwards, remove the media from each well and add 180 μL of fresh media.

**3.4.3** Add 20 μL of fluorescently labeled compounds (FITC-dextran or fluorescently labeled compounds of choice) to each well, resulting in a final concentration of 0.1 – 1 μM. Prepare single stain controls following the same steps as the experimental groups by treating the cells with fluorescently labeled compounds at a higher concentration (5~10X). Exclude the staining by Aqua zombie Live/Dead solution specified in **Step 3.4.8** for single stained controls. For unstained control or live/dead control cells, add 20 μL of culturing media to each well. Prepare all experimental groups in triplicates, except for flow compensation groups.

**3.4.4** Carefully remove the media from each well after incubation at 37°C for 4 hr. Wash the cells once with 200 μL of PBS, and then add 100 μL of trypsin to each well. Allow trypsinization at 37°C for 5 min until most of the cells detach. Neutralize trypsin activity by adding 100 μL of media to each well.

**3.4.5** Gently pipette the cells up and down in each well and transfer them to either a 96-well V-bottom plate or 1.5 mL microcentrifuge tubes. Spin down the cells at 1,700 rpm (271 rcf) for tubes using a microcentrifuge, or 1,700 rpm (578 rcf) for plates using a benchtop centrifuge for 5 min [Note 1].

**3.4.6** Remove the supernatant and add 200 μL of PBS. Spin down the cells again at 1,700 rpm for 5 min and remove the supernatant.

**3.4.7** For unstained control and single stained control, continue from **Step 3.4.10** onwards. For the live/dead control, add 50 μL of 80% ethanol and incubate at RT for 5 min. Add 150 μL of PBS, spin down the cells, and remove the supernatant. Proceed with steps from **Step 3.4.8** for the live/dead control and experimental groups. Add a small aliquot of fixed unstained cells to the fixed live/dead control before flow analysis.

**3.4.8** Add 100 μL of Aqua zombie Live/Dead solution (1:200 diluted in 1X PBS) to each well on the plate or each tube to resuspend the pellets. Keep the cells on ice in the dark for 20 min, then spin down at 1,700 rpm for 5 min.

**3.4.9** Remove the supernatant, add 200 μL of PBS, and spin down the cells at 1,700 rpm for 5 min. Remove the supernatant.

**3.4.10** Resuspend the cell pellets in 100 μL of 4% PFA and allow fixation at RT for 15 min. Spin down the cells at 1,700 rpm for 5 min.

**3.4.11** Remove the supernatant, add 200 μL of PBS, and spin down the cells at 1,700 rpm for 5 min. Remove the supernatant, add 200 μL of PBS for resuspension, and transfer the cell suspension to a FACs tube for flow cytometry analysis.

*Note 1: The maximum volume loaded for a well on a 96-well V-bottom plate should be less than 200 μL for optimal working efficiency. If a plate instead of a tube is used for cell collection, add 100 μL media to neutralize trypsin.*

### 3.5 DC maturation protocol

**3.5.1** Seed 4.5x10^4^ cells in each well of a 48-well plate, followed by topping up with culturing media to 1.0 mL.

**3.5.2** Remove the media after incubation in an incubator supplemented with 5% CO_2_ at 37 °C overnight (~18 hr), followed by adding 900 μL media to each well.

**3.5.3** Dissolve 100 μL peptide vaccines in 1X PBS and add it to each well to make a final antigen concentration of 10 μM in 1 mL media [Note 2]. In addition, use 20 ng IFN-γ, 1.0 μg lipopolysaccharide (LPS) or 1.0 μg Pam_2_CSK_4_ per well in certain groups to serve as positive controls [Note 3]. For cells used as unstained, live/dead, or FMO controls, add 100 μL PBS instead of antigen solution in each well. Perform triplicates for all groups. Allow the cells to be activated by the vaccines for 24 hr in the incubator at 37 °C.

**3.5.4** Remove the media, and wash the cells with 200 μL PBS once, then add 100 μL trypsin to each well. Allow trypsinization for 5 min in an incubator at 37 °C, then add 100 μL media to neutralize trypsin activity in each well. Mix the cells in each well by pipetting up and down gently, and then transfer the cell suspensions to a 96-well V-bottom plate or 1.5 mL microcentrifuge tubes, followed by spinning down the cells using a benchtop centrifuge for plates at 1,700 rpm (578 rcf), or a microcentrifuge for tubes at 1,700 rpm (271 rcf) for 5 min. Remove the supernatant and resuspend the cells in 200 μL of sterile PBS. Spin down the cells at 1,700 rpm for 5 min, then remove the supernatant.

**3.5.5** For unstained control, perform **Step 3.5.10**; for live/dead control, add 50 μL of 80% ethanol and incubate it for 5 min at RT, and add 150 μL PBS, then spin down the cells and aspirate the supernatant. Re-suspend the cells of live/dead control in 100 μL Aqua zombie dye (1:200 diluted in 1X PBS) for incubation on ice in the dark for 20 min. For live/dead control, perform **Step 3.5.9** onwards to fix live/dead control after washing the cells once. Add a small aliquot of fixed untreated live cells to fixed live/dead control before flow analysis.

**3.5.6** Re-suspend all experimental groups except for the abovementioned groups in **Step 3.5.5** (unstained and live/dead control) in 100 μL TruStain FcX™ solution (1:200 diluted in 1X PBS) [Note 4]. Allow incubation on ice in the dark for 25 min. Spin down the cells at 1,700 rpm for 5 min, and then remove the supernatant.

**3.5.7** Wash the cells by adding 200 μL PBS. Spin down the cells at 1,700 rpm for 5 min, and discard the supernatant, then re-suspend the cells in 100 μL Aqua zombie Live/Dead (1:200 diluted in PBS) [Note 4]. Allow incubation on ice in the dark for 20 min.**3.5.8** Wash the cells by adding 200 μL PBS. Spin down the cells at 1,700 rpm for 5 min, and discard the supernatant. Re-suspend the cells designated for experimental groups in 100 μL antibody cocktail consisting of PE anti-mouse CD40 (anti-CD40), APC-Cy7 anti-mouse I-A/I-E (anti-MHC-II), FITC anti-mouse CD80 (anti-CD80) and BV421 anti-mouse CD86 (anti-CD86) (all 1:200 diluted in PBS) [Note 4]. Re-suspend cells designated to be FMO1 in 100 μL FMO1 (CD40 FMO) cocktail consisting of anti-MHC-II, anti-CD80 and anti-CD86 (1:200 diluted in PBS); re-suspend cells designated to be FMO2 in 100 μL FMO2 (MHC-II FMO) cocktail consisting of anti-CD40, anti-CD80 and anti-CD86 (1:200 diluted in PBS); re-suspend cells designated to be FMO3 in 100 μL FMO3 (CD80 FMO) cocktail consisting of anti-CD40, anti-MHC-II and anti-CD86 (1:200 diluted in PBS); re-suspend cells designated to be FMO4 in 100 μL FMO4 (1:200 diluted in PBS) cocktail [Note 3]. Allow incubation on ice in the dark for 25 min.

Meanwhile, prepare single stained bead controls by mixing 1.0 μL anti-CD40, anti-MHC-II, anti-CD80, or anti-CD86 with a drop of positive bead and a drop of negative bead from the Igκ bead kit. Allow incubation at RT in the dark for 15 min.

**3.5.9** Spin down the cells or beads at 1,700 rpm for 5 min, remove the supernatant, then add 200 μL of PBS. Repeat the washing step once before the next step.

**3.5.10** Re-suspend the cells or beads in 100 μL 4% PFA for fixation at RT for 15 min [Note 4] [Note 5], and then spin down at 300 g for 5 min. Wash the cells or beads with 200 μL PBS once before re-suspension in 200 μL PBS, and then transfer them into a FACs tube for flow cytometry analysis.

*Note 2: The compound concentration is subject to change depending on the inherent properties of compounds to be tested. This is crucial as a high concentration of certain compounds may lead to cell toxicity or signal saturation, potentially skewing the experimental results. In cases where signal saturation is observed, particularly when analyzed by frequency (the percentage of cells exhibiting a specific fluorochrome signal), it is advised to use the median fluorescence intensity (MFI) as an alternative measure. MFI provides a more accurate representation of the signal intensity per cell.*

*Note 3: To maintain the integrity of reagents and compound solutions, it is imperative to minimize freeze-thaw cycles. For compounds exhibiting limited aqueous solubility, the utilization of dimethyl sulfoxide (DMSO) is recommended at a low concentration. For instance, a 4% (v/v) concentration of DMSO has been demonstrated to be sufficient for dissolving hydrophobic compounds in stock solutions used in this study. Compounds should be first be dissolved in the predetermined volume of DMSO, followed by dilution with PBS. Additionally, if DMSO is employed in any experimental group, it is crucial to include an equivalent concentration of DMSO in every other group to ensure experimental consistency.*

*Note 4: The volume of reagents used as indicated is tied to the number of cells being treated. For example, if cells from two separate wells are to be combined for a staining procedure, it is important to double the volume of reagents used in the procedure.*

*Note 5: Fixation can be waived if cells will be analyzed by flow shortly after staining. Fixed cells should be kept in a refrigerator at 4°C in the dark for short-term storage. It is also important to note that beads should also be fixed if cells are fixed.*

### 3.6 Flow cytometry analysis

The acquisition of flow cytometry events was carried out using BD LSRFortessa™ X-20 Cell Analyzer with BD FACSDiva software (BD Biosciences). The compensation set-up was conducted by using the compensation beads stained with single-colored fluorescent-conjugated antibodies. After the compensation set-up, the events for full stained samples and FMO samples were acquired. At least 10,000 events were recorded for each of the samples. Finally, the data were exported as FCS files and analyzed using FlowJo™ v10.8 software (BD Life Sciences).

## 4 Results

### 4.1 Characterization of the surface marker expression of DC2.4 cells

**Figure 1:**
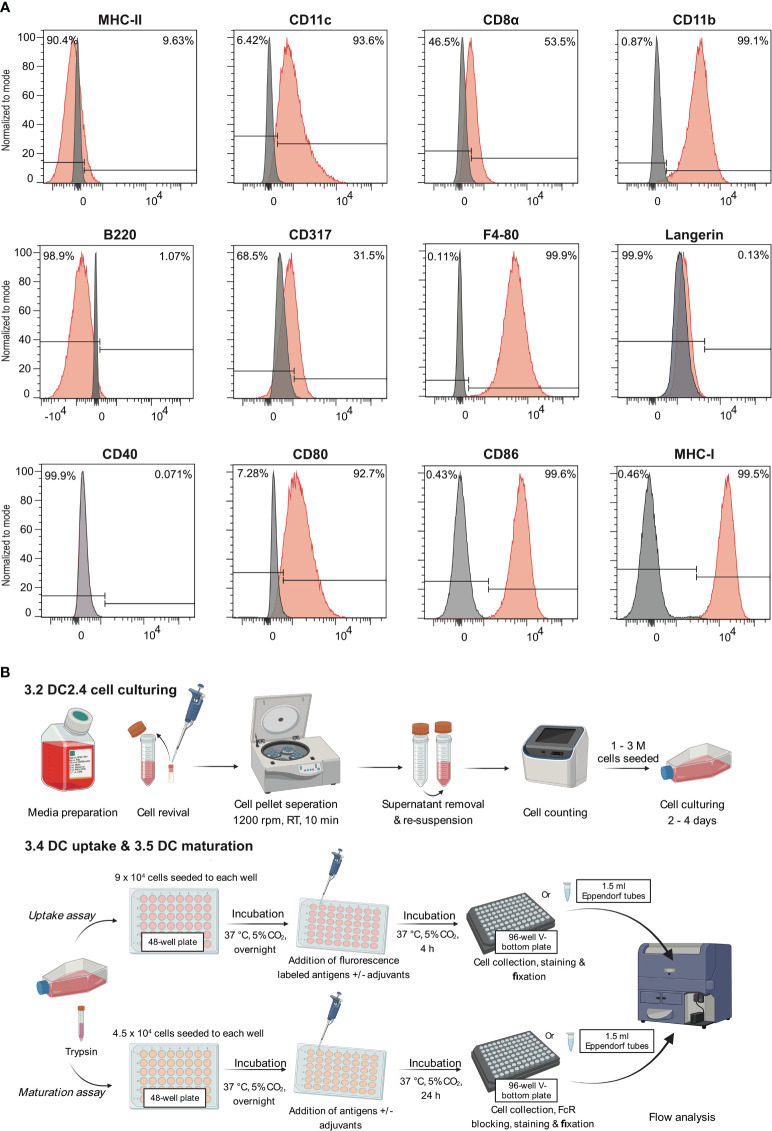
Leveraging the semi-mature state of DC2.4 cells to evaluate the efficacy of adjuvants. **(A)** Characterization of cell surface markers on DC2.4 cell line by flow cytometry. The grey histogram represents the fluorescence signal of the control samples (FMO control), and the red histogram represents the fluorescence of a given marker in the sample. **(B)** Schematics of *in vitro* DC2.4 cell uptake and maturation assays.

The expression of markers including CD11c, CD11b, F4/80, MHC-I, MHC-II, CD8α, CD317, B220, CD80, CD86, and CD40, were analyzed by flow cytometry (**Figure 1A**; **Supplementary Figure S1**). The analysis of these surface markers revealed a distinct expression profile on DC2.4 cells, characterized by high levels of CD11c, CD11b, F4/80, MHC-I, CD80, CD86, moderate expression of CD8α and CD317, and low to no expression of MHC-II, B220 and CD40. This expression pattern is consistent with previous studies (10), which have suggested that the DC2.4 cell line exhibits a semi-mature phenotype in its resting state, characterized by high expression of MHC-I, CD80, and CD86. Notably, while these cells inherently express high levels of CD80 and CD86 (15), their expression can be further amplified upon activation with certain stimulants (16). Consequently, CD80 and CD86 serve as pivotal markers for assessing the maturation status of DC2.4 cells. This assessment is complemented by tracking markers such as CD40 and MHC-II, which exhibit low to negligible expression in the absence of stimulation.

### 4.2 DC2.4 cell FITC-dextran uptake assay

**Figure 2:**
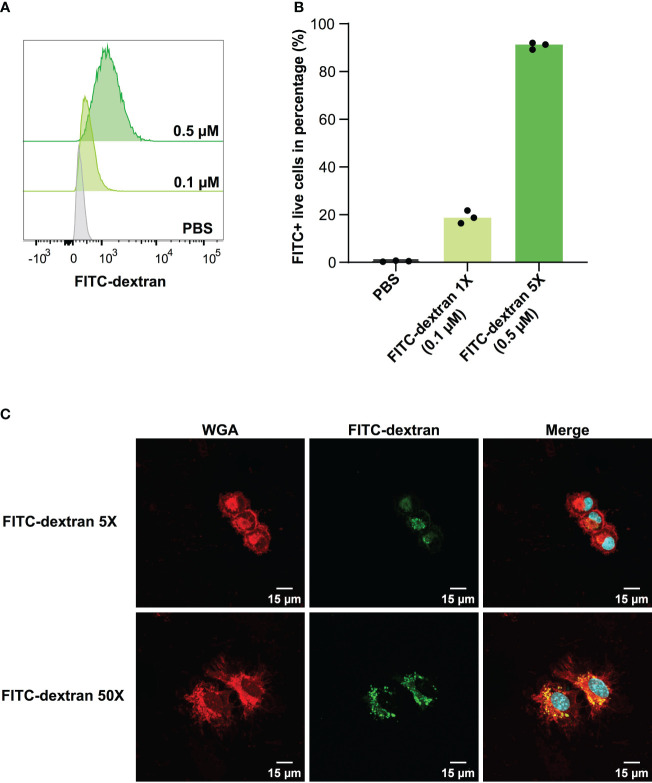
Concentration-dependent uptake of FITC-dextran by DC2.4 cells. **(A)** Mean fluorescent Intensity of the uptake of 0.1 and 0.5 μM FITC-dextran by DC2.4 cells. **(B)** Percentage of dextran positive DC2.4 cells post uptake of 0.1 and 0.5 μM FITC-dextran by DC2.4 cells. **(C)** Confocal microscopy images of DC2.4 cells after co-incubation with 0.5 (*top*) or 5 (*bottom*) μM FITC-dextran for 4 h (20X objective). Red channel: wheat germ agglutinin (WGA) labeling cell membrane; Blue channel: 4’,6-diamidino-2-phenylindole (DAPI) labeling nucleus; Green channel: FITC labeling dextran internalized by DC2.4.

In the context of vaccine development, the efficacy of a vaccine is largely dependent on the successful presentation of antigens by DCs to T cells. Adjuvants that enhance antigen uptake by DCs have the potential to substantially increase the likelihood of antigens being presented. Considering this, we evaluated the capacity of DC2.4 cells to uptake fluorescent dextran (FITC-tagged) via pinocytosis (17) by flow cytometry (**Figure 2**). The results demonstrated that DC2.4 cells displayed concentration-dependent uptake of FITC-dextran. To prevent signal saturation in future uptake assays, a concentration range of 0.1 – 0.5 μM was identified as suitable (**Figures 2A, B**). Using confocal microscopy, we confirmed the presence of FITC-dextran mainly cytoplasmatic granules following a 4-hour incubation with DC2.4 cells (**Figure 2C**, *top*). This localization became even more pronounced when FITC-dextran was administered at a concentration ten times higher (5 μM) to DC2.4 cells (**Figure 2C**, *bottom*).

Furthermore, we synthesized a cyanine5.5-tagged peptide antigen (Cy5.5-PADRE-J8) and assessed its uptake by DC2.4 cells at a concentration of 1 μM (**Supplementary Information**). Flow cytometry analysis demonstrated a robust Cy5.5 signal intensity, indicating effective internalization of Cy5.5-PADRE-J8 in DC2.4 cells. It’s essential to ensure a distinct difference in signal intensity between unadjuvanted antigens and those with adjuvants for accurate observations. Therefore, conducting compound titration is always imperative (**Supplementary Figure 2**).

### 4.3 DC maturation and cytokine production

**Figure 3:**
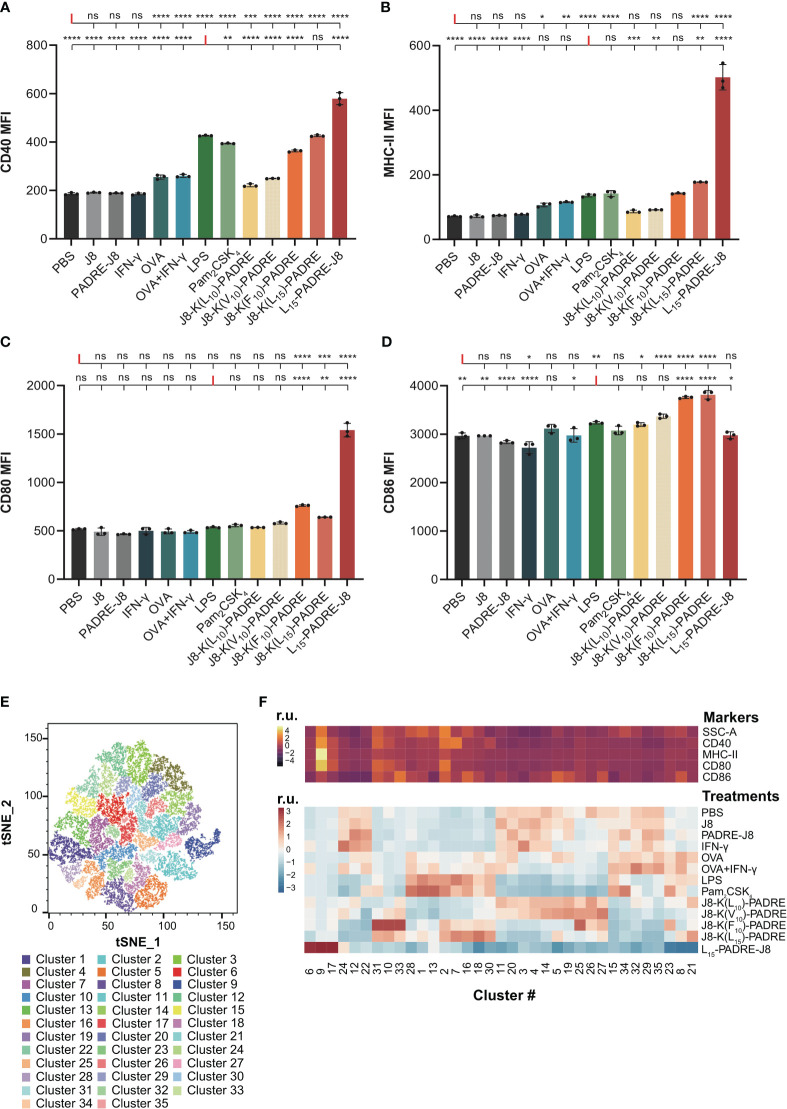
Maturation of DC2.4 cells by peptide vaccines. **(A)** MFI of MHC-II expression in live DC2.4 cell population post-vaccine treatment. **(B)** MFI of CD40 expression in live DC2.4 cell population post-vaccine treatment. **(C)** MFI of CD80 expression in live DC2.4 cell population post-vaccine treatment. **(D)** MFI of CD86 expression in live DC2.4 cell population post-vaccine treatment. **(E)** Clustering of different vaccines-treated DC2.4 cells by SSC, and expression of CD40, MHC-II, CD80 and CD86. **(F)** Top heatmap depicting the relative SSC, and expression of CD40, MHC-II, CD80 or CD86, measured in median fluorescent intensity. Bottom heatmap represents the percentage distribution of cells treated with different groups within each cluster. r.u. relative units. *: p-value < 0.05; **: p-value < 0.01; ***: p-value < 0.001; ****: p-value < 0.0001; "ns" means 'not significant'.

After the evaluation of key DC markers, we selected CD40, MHC-II, CD80 and CD86 to evaluate the maturation status of DC2.4 cells in our assay (**Figure 1A**). Informed by the literature, LPS and a mixture of OVA and IFN-γ were employed as the positive controls to activate DC2.4 cells (18, 19). The inclusion of IFN-γ was substantiated by its well-established role as a CD40 inducer (20). We also used a robust toll-like receptor (TLR) 2/6 agonist, Pam_2_CSK_4_ as a positive treatment control. Furthermore, the potential of peptide antigens conjugated to peptide-based adjuvants (specially L_15_-PADRE-J8, J8-K(L_15_)-PADRE, J8-K(L_10_)-PADRE, J8-K(F_10_)-PADRE, and J8-K(V_10_)-PADRE) in upregulating the selected markers was evaluated. This potential was then compared with that of naked antigens, namely J8, which acts as a B-cell epitope, and PADRE-J8, where PADRE (pan HLA-DR epitope) functions as a T helper epitope (**Figures 3A–D**; **Supplementary Figure 7**).

The expression of CD40 was significantly increased in all groups except for cells treated with PBS, J8, PADRE-J8 and IFN-γ (**Figure 3A**). Notably, the lead candidate, L_15_-PADRE-J8, which showed the most promising results in animal studies by eliciting the highest antigen-specific IgG titers, also significantly upregulated CD40 expression, surpassing even that induced by LPS. Akin to PBS, J8, PADRE-J8 and IFN-γ, J8-K(L_10_)-PADRE and J8-K(V_10_)-PADRE failed to upregulate the expression of MHC-II (**Figure 3B**). Not surprisingly, L_15_-PADRE-J8 induced the highest level of MHC-II expression, followed by J8-K(L_15_)-PADRE. This outcome aligned well with our previous animal studies, where both L_15_-PADRE-J8 and J8-K(L_15_)-PADRE demonstrated exceptional promises. In those studies, these two compounds effectively stimulated the production of protective sera against the targeted bacterium (13, 21). In addition to CD40 and MHC-II, L_15_-PADRE-J8 significantly increased the expression of CD80 on the surface of DC2.4 cells. Interestingly, the remaining groups, except J8-K(F_10_)-PADRE and J8-K(L_15_)-PADRE, did not exhibit notable upregulation of CD80 (**Figure 3C**). However, L_15_-PADRE-J8 failed to upregulate CD86 expression, whereas LPS, Pam_2_CSK_4_ and other poly(hydrophobic amino acids) derivatives (J8-K(V_10_)-PADRE, J8-K(F_10_)-PADRE, and J8-K(L_15_)-PADRE) significantly increased it (**Figure 3D**). The difference in CD80/86 expression patterns amongst groups indicated different maturation statuses of cells stimulated by different compounds. L_15_-PADRE-J8 may have led to a more advanced maturation status in DC2.4 cells. No significant differences were observed among groups when analyzing the frequency of CD86 expression in DC2.4 cells (**Supplementary Figure 4**).

To discern between different levels of maturation, we conducted unbiased clustering of all treatment groups using FlowJo’s tools, Phenograph and Cluster Explorer (**Figures 3E, F**). This clustering was based on the physical parameter Side-scatter (SCC), which serves as a proxy for cell granularity, and the median intensity of CD40, MHC-II, CD80 and CD86 expression. In this analysis, we identified a total of thirty-five distinct clusters across the various treatment groups (**Figure 3E**). Cells treated with the lead compound, L_15_-PADRE-J8, were mainly clustered in clusters 6, 9 and 17 (**Figure 3F**). The SSC and the expression of CD40, MHC-II and CD80 of cluster 9 were significantly higher than other clusters, suggesting a distinctive marker signature of cells treated with L_15_-PADRE-J8. Cells treated with PBS, J8, PADRE-J8 and IFN-γ exhibited similar cluster distribution. These clusters (3, 4, 11, 23, 14, 15, 20, 22, 24, 29, 32 and 35) all showed low expression of CD40, MHC-II and CD80. Interestingly, cells treated with OVA or OVA+IFN-γ, LPS or Pam_2_CSK_4_, J8-K(L_10_)-PADRE or J8-K(V_10_)-PADRE, and J8-K(F_10_)-PADRE or J8-K(L_15_)-PADRE showed similar cluster distribution, respectively.

**Figure 4:**
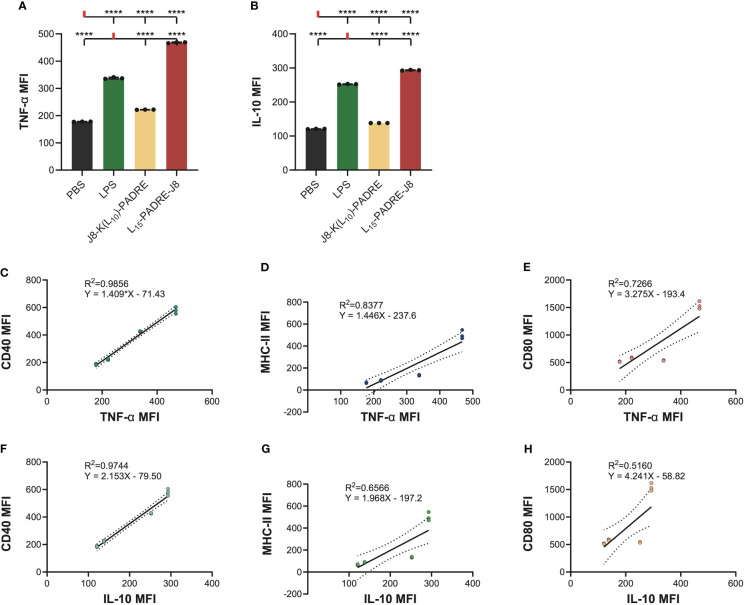
Intracellular cytokine staining of cells treated with PBS, LPS, J8-K(L_10_)-PADRE, and L_15_-PADRE-J8. **(A)** TNF-α MFI of cells treated with PBS (shown in black), LPS (shown in green), J8-K(L_10_)-PADRE, and L_15_-PADRE-J8 (shown in red). **(B)** IL-10 MFI of cells treated with PBS (shown in black), LPS (shown in green), J8-K(L_10_)-PADRE, and L_15_-PADRE-J8 (shown in red). **(C)** Linear regression model of TNF-α MFI and CD40 MFI. **(D)** Linear regression model of TNF-α MFI and MHC-II MFI. **(E)** Linear regression model of TNF-α MFI and CD80 MFI. **(F)** Linear regression model of IL-10 MFI and CD40 MFI. **(G)** Linear regression model of IL-10 MFI and MHC-II MFI. **(H)** Linear regression model of IL-10 MFI and CD80 MFI. ****: p-value < 0.0001.

Since DCs secrete cytokines to crosstalk with T cells to regulate downstream immune responses, we measured the intracellular accumulation of two key cytokines, a pro-inflammatory cytokine, tumor necrosis α (TNF-α), and an anti-inflammatory cytokine, interleukin-10 (IL-10) (**Supplementary Information, Supplementary Figure S5**). As expected, L15-PADRE-J8 elicited robust production of both TNF-α and IL-10, surpassing the response induced by LPS (**Figures 4A, B**). This suggests a potent inflammatory reaction, potentially counterbalanced by an anti-inflammatory response to maintain homeostasis. Conversely, J8-K(L_10_)-PADRE only weakly induced the production of TNF-α. To elucidate the relationship between the expression patterns of CD40, MHC-II, CD80, or CD86 and that of TNF-α or IL-10 during DC activation, linear regression models were used (**Figures 4C–H**). The analysis revealed a high correlation between the expression of TNF-α and CD40, TNF-α and MHC-II, or IL-10 and CD40, with R2 values of 0.9856, 0.8377, and 0.9744, respectively (**Figures 4C, D, F**). Furthermore, a moderate correlation was observed between the expression of TNF-α and CD80, IL-10 and MHC-II, or IL-10 and CD80, with R2 values of 0.7266, 0.6566, and 0.5160 (**Figures 4E, G, H**). However, no linear correlation was found between the expression of TNF-α and CD86, or IL-10 and CD86 (**Supplementary Figure S6**).

## 5 Discussion

In vaccine development, the implementation of both *in vivo* and *in vitro* studies is essential. *In vivo* studies provide direct data on the safety and efficacy of vaccines, while *in vitro* studies aim to elucidate the mechanisms of vaccine effectiveness. Employing predictive *in vitro* assays before *in vivo* studies aids in the optimization of vaccine development by identifying preparations that are likely to elicit robust immune responses, thus reducing the utilization of ineffective vaccines in animal experimentation. Both DCs and macrophage cell lines, including RAW264.7 and J774 (22), have been extensively utilized as APCs in research. Here, we present a DC-based assay that has the potential to predict the efficacy of peptide-based vaccines in subsequent *in vivo* studies.

We employed an experimental design where subunit vaccines were directly internalized by DC2.4 cells. We conducted two experiments to evaluate the internalization of candidate subunit vaccines by DCs, and their ability to upregulate DC maturation markers. At first, we investigated DC uptake, employing a quantitative method to analyze internalization using fluorescein-tagged peptide antigens. It is important to note that the use of different fluorescein tags on the same antigen may result in varying levels of DC uptake, even at identical concentrations; namely, the fluorescent tag of choice can influence uptake (23).

DC2.4 cells (*Merck repository*) naturally showed relatively high expression of CD86 and MHC-I at the resting state, but low expression of CD40 and MHC-II. Therefore, we selected MHC-II and several co-stimulatory markers including CD40, CD80, and CD86 to elucidate the potential of vaccine candidates to induce DC2.4 cell maturation. Although IFN-γ is a known CD40 inducer, the addition of IFN-γ (20 ng/mL) did not upregulate CD40 expression in DC2.4 cells (**Figure 3A**). This could be attributed to a different maturation state of our cells purchased from Merck. Several peptide vaccine constructs, previously shown to induce different magnitudes of humoral responses *in vivo*, were employed to stimulate DC2.4 cells. J8-K(V_10_)-PADRE, J8-K(F_10_)-PADRE, and J8-K(L_10_)-PADRE, which were previously reported to be less immunogenic (13, 21), stimulated DC2.4 cells to a less extent compared to stronger peptide vaccine constructs, J8-K(L_15_)-PADRE and L_15_-PADRE-J8. Lower CD40, MHC-II, and CD80 expression was found in cells treated with J8-K(V_10_)-PADRE, J8-K(F_10_)-PADRE, or J8-K(L_10_)-PADRE compared with cells treated with J8-K(L_15_)-PADRE or L_15_-PADRE-J8. Interestingly, all poly(hydrophobic amino acid)-adjuvanted antigens (J8-K(V_10_)-PADRE, J8-K(F_10_)-PADRE, J8-K(L_10_)-PADRE, and J8-K(L_15_)-PADRE) except for L_15_-PADRE-J8 upregulated CD86 expression compared to PBS, indicating a different maturation state of cells treated with L_15_-PADRE-J_8_. Studies have shown that dendritic cells express both CD80 (B7.1) and CD86 (B7.2) upon activation. CD86 is recognized as a marker for early maturation, whereas CD80 expression typically increases only in fully mature DCs (24). In fact, CD80 has a higher monomeric affinity for CD80/86’s ligands, CD28 or CTLA-4, than CD86 (25). While we cannot disregard the potential impact of DC-trafficking and interactions with other cells that take place *in vivo*, the notable strong correlation observed between our *in vitro* assay and *in vivo* studies bolsters the validity of our model as a valuable preliminary screening tool before animal experimentation.

The subsequent cytokine profiling indicated that J8-K(L_10_)-PADRE was weaker in generating pro-inflammatory responses than L_15_-PADRE-J8. Interestingly, we found that L_15_-PADRE-J8 not only strongly induced the production of pro-inflammatory cytokine, TNF-α, but also anti-inflammatory cytokine, IL-10. This intricate balance facilitated by TNF-α and IL-10 highlights a sophisticated feedback loop within dendritic cells, essential for modulating the immune system’s response to ensure an equilibrium between pro-inflammatory and anti-inflammatory signals (26). In our linear regression models, we found that the correlation between CD40 and TNF-α or IL-10 expression was particularly strong (R^2 =^ 0.9856 and 0.9744, respectively). This implies that CD40 may serve as a reliable marker for indicating the production levels of TNF-α and IL-10. As previously reported, all poly(hydrophobic amino acids)-containing vaccine formulations (J8-K(V_10_)-PADRE, J8-K(F_10_)-PADRE, J8-K(L_10_)-PADRE, J8-K(L_15_)-PADRE, and L_15_-PADRE-J8) self-assemble into nanoparticles (13, 21, 27, 28), which facilitate their recognition by APCs, including DCs, thereby enhancing the internalization of vaccine components and further accelerating the processing of antigens and presentations by DCs. This was in line with our findings that naked antigen (J8, or PADRE-J8) without a nanoparticulate delivery system could not upregulate CD40, MHC-II, CD80 or CD86 expression on DC2.4 cells.

The method proposed here offers a simplified approach for swiftly and affordably screening vaccine formulations, facilitating subsequent validation steps. To confirm the successful induction of antigen-specific T cells, our method can be complemented with tetramer staining. This allows for the detection of antigen-specific T cells following the exposure of vaccine-activated dendritic cells. In the presented method, we have optimized different variables related to DC2.4 culturing and passaging to ensure both high reproducibility and straightforward applicability. For instance, we optimized the seeding density and increased the frequency of media changes to once per day. These modifications resulted in accelerated cell growth and consistently high cell viability, exceeding 97% at the time of collection. Moreover, for uptake and maturation studies, we determined that the appropriate cell confluency was 80% to prevent over-confluency and cell death. Both LPS and OVA were suitable positive controls for maturation.

Additionally, we recommend the use of V-bottom plates as they minimize cell loss during washing steps. We meticulously optimized compound concentrations to prevent signal saturation, providing a guideline for future experiments that will avoid signal saturation and ensure differentiation between experimental groups. Of note, antibody titration should be performed when determining the most appropriate concentration of antibodies for staining, which is subject to change when activation compounds perform differently in upregulating certain markers.

It is important to underscore the versatility of the presented assay, as it enables the evaluation of not only peptide-based vaccines but also protein-based vaccines or standalone adjuvants upon careful titration of antigen/adjuvant concentrations.

## 6 Conclusion

Here, we present a refined protocol to test the capacity of synthetic vaccines to induce DC maturation *in vitro*. Our comprehensive methods offer essential insights into the ideal seed cell density, compound concentration, incubation duration, and staining protocols. These details are instrumental in achieving a reproducible, efficient, and high-throughput assessment of subunit vaccine candidates.

## Data availability statement

The original contributions presented in the study are included in the article/**Supplementary Material**. Further inquiries can be directed to the corresponding authors.

## Ethics statement

Ethical approval was not required for the studies on animals in accordance with the local legislation and institutional requirements because only commercially available established cell lines were used.

## Author contributions

LL: Data curation, Formal analysis, Investigation, Methodology, Validation, Visualization, Writing – original draft, Writing – review & editing. WK: Data curation, Formal analysis, Investigation, Methodology, Software, Validation, Visualization, Writing – review & editing. JZ: Data curation, Investigation, Methodology, Validation, Writing – review & editing. FF: Data curation, Investigation, Methodology, Writing – review & editing. JW: Resources, Supervision, Writing – review & editing. RS: Resources, Supervision, Writing – review & editing. IT: Funding acquisition, Resources, Supervision, Writing – review & editing. MS: Conceptualization, Funding acquisition, Methodology, Project administration, Resources, Supervision, Writing – review & editing. JC: Conceptualization, Data curation, Formal analysis, Funding acquisition, Investigation, Methodology, Project administration, Resources, Software, Supervision, Validation, Visualization, Writing – original draft, Writing – review & editing.
