# Supplementary material for: Utilizing murine dendritic cell line DC2.4 to evaluate the immunogenicity of subunit vaccines in vitro
Source: Front Immunol. 2024 Feb 26;15:1298721. doi: 10.3389/fimmu.2024.1298721 (PMC10925716; doi:10.3389/fimmu.2024.1298721)
Supplement: Supplementary file 1 [file DataSheet_1.docx]

**Supplementary Information**

Utilizing murine dendritic cell line DC2.4 to evaluate the immunogenicity of peptide vaccines *in vitro*

Lantian Lu^1,2^, Wei Yang Kong^2^, Jiahui Zhang^1,2^, Farrhana Firdaus^1^, James W. Wells^2^, Rachel J. Stephenson^1^, Istvan Toth^1,3,4^, Mariusz Skwarczynski^1, *^, Jazmina L. Gonzalez Cruz^2, *^

^1^ School of Chemistry and Molecular Biosciences, The University of Queensland, St. Lucia, QLD 4072, Australia

^2^ Faculty of Medicine, Frazer Institute, The University of Queensland, Woolloongabba, QLD 4102, Australia

^3^ Institute of Molecular Bioscience, The University of Queensland, St. Lucia, QLD 4072, Australia

^4^ School of Pharmacy, The University of Queensland, Woolloongabba, QLD 4102, Australia

*** Correspondence:**Jazmina Gonzalez Cruz; Mariusz Skwarczynski
[j.gonzalezcruz@uq.edu.au](mailto:j.gonzalezcruz@uq.edu.au); [m.skwarczynski@uq.edu.au](mailto:m.skwarczynski@uq.edu.au)

## Synthesis of Cyanine5.5-tagged PADRE-J8 peptide

PADRE-J8 was synthesized using Boc-based solid-phase peptide synthesis as described in Section 3 Methods. Azide-linked PADRE-J8 (N_3_-PADRE-J8) was synthesized by coupling azido-acetic acid (4.2 eq.), with the help of hexafluorophosphate azabenzotriazole tetramethyl uronium (HATU; 4 equivalence) and *N,N*-diisopropylethylamine (DIPEA; 6.2 equivalence), to the N-terminus of PADRE-J8 on rink amide MBHA resin. Fluorophore Cy5.5 was tagged to PADRE-J8 through copper(I) catalyzed alkyne-azide 1,3-dipolar cycloaddition (CuCCA) click reaction as described previously [1].

In brief, the pure N_3_-PADRE-J8 (4.0 mg, 0.00085 mmol, 1.0 eq.) was dissolved in MeOH/H_2_O solution (5.0 mL, 50% v/v) and mixed with a solution of Cy5.5-alkyne (0.635 mg, 10 µmol, 1.2 eq.) in DMSO (10 μL, 10 mM). Following the addition of CuSO_4_ (1.5 mg, 8.8 equivalence) and NaAsc (3.0 mg, 18 eq.), the reaction was stirred at 50 ⁰C for 2 hrs and monitored by analytical RP-HPLC and ESI-MS. Upon completion of the reaction, two drops of TFA were added to the reaction mixture until the suspension disappeared. The crude Cy5.5-PADRE-J8 was obtained and purified by preparative RP-HPLC (C18 column, at a flow rate of 20 mL/min) with a gradient of 40% solvent B to 70% solvent B, yielding a blue solid.

**Cy5.5-PADRE-J8**. Yield: 70%. Molecular weight: 5315.29 g/mol. ESI-MS [M + 3H]^3+^ m/z 1772.6 (calcd: 1772.8), [M + 4H]^4+^ m/z 1330.3 (calcd: 1329.8), [M + 5H]^5+^ m/z 1064.5 (calcd: 1064.1 ), [M + 6H]^6+^ m/z 886.9 (calcd: 886.9), [M + 7H]^7+^ m/z 760.6 (calcd: 760.3), [M + 8H]^8+^ m/z 665.7 (calcd: 665.4), [M + 9H]^9+^ m/z 591.8 (calcd: 591.6), [M + 10H]^10+^ m/z 532.3 (calcd: 532.5). t_R_ = 29.7 min (0-100% solvent B; C18 column); purity ≥ 99%.

**Figure S0a.** Mass spectrometric analysis of Cy5.5-PADRE-J8.

**Figure S0b.** HPLC analysis of Cy5.5PADRE-J8

## Confocal Microscopy Imaging

Cells for confocal microscopy were cultured in a 24-well plate. In brief, 15x10^4 cells were seeded per well in a 24-well plate with a coverslip placed at the bottom before cell seeding. Incubation was performed the same as described in the manuscript (Section **3.4**). Post overnight incubation, the cells were treated with PBS, 0.5 μM and 5 μM FITC-dextran, respectively. The media was discarded after 4 h incubation, followed by extensive washing with PBS, and fixation with 1 mL 4% PFA at 37 ℃ for 15 min. The cells were then stained with 100 μL staining solution containing 0.5 μg Wheat Germ Agglutinin (WGA) and 0.1 μg DAPI and allowed incubation at RT for 30 min. Finally, the cells were mounted onto a glass slide using mounting media and dried overnight in a refrigerator before confocal microscopy imaging.

Images were taken using Olympus FV3000 microscope with a 20X objective lens, and saved in OIR files for analysis using Fiji.

## Intracellular Cytokine Staining

- Seed 4.5x10^4^ cells in each well of a TC-treated 48-well plate, followed by topping up with culturing media to 1.0 mL.
- Remove the media after incubation in an incubator supplemented with 5% CO_2_ at 37 ℃ overnight (~18 hr), followed by adding 900 μL media to each well.
- Dissolve 100 μL peptide vaccines in 1X PBS and add it to each well to make a final antigen concentration of 20 μM in 1 mL media or add LPS to make a concentration of 1.0 μg/mL. Allow incubation with 5% CO_2_ at 37 ℃ for 24 h [Note 1].
- Remove the media, and wash the cells with 200 μL PBS once, then add 100 μL trypsin to each well. Allow trypsinization for 5 min in an incubator at 37 ℃, then add 100 μL media to neutralize trypsin activity in each well. Mix the cells in each well by pipetting up and down gently, and then transfer the cell suspensions to a 96-well V-bottom plate, followed by spinning down the cells using a benchtop centrifuge for plates at 1,700 rpm (578 rcf), for 5 min. Remove the supernatant and resuspend the cells in 200 μL of sterile PBS. Spin down the cells at 1,700 rpm for 5 min, then remove the supernatant.
- Add 100 μL Aqua zombie Live/Dead solution (1:200 in PBS) to each well and resuspend the cells. Allow incubation on ice in the dark for 25 min. Spin down the cells at 1,700 rpm for 5 min and then remove the supernatant.
- Wash the cells with 200 μL PBS, and spin down the cells at 1,700 rpm for 5 min, then remove the supernatant. Resuspend cells in 100 μL Trustain FcX^TM^ solution (1:200 diluted in PBS) and allow incubation on ice in the dark for 25 min.
- Spin down the cells at 1,700 rpm for 5 min. Add 200 μL PBS to wash the cells, and spin down the cells again at 1,700 rpm for 5 min, then discard the supernatant.
- Resuspend cells in 100 μL 1X fixation & permeabilization buffer (fixation/permeabilization concentrate 1:4 diluted in eBioscience^TM^ fixation/permeabilization diluent), and allow incubation at RT for 20 min. Add 100 μL 1X permeabilization buffer (permeabilization buffer 10X 1:10 diluted in MilliQ water) to cells, and then spin down the cells at 1,700 rpm at RT for 5 min.
- Discard the supernatant, and resuspend cells in 200 μL 1X permeabilization buffer, then spin down the cells at 1,700 rpm at RT for 5 min.
- Discard the supernatant, and resuspend cells in 50 μL antibody cocktails (BV785 anti-mouse TNF-α and PE anti-mouse IL-10 1:50 diluted in 1X permeabilization buffer), then allow incubation at RT for 20 min. Add 100 μL 1X permeabilization buffer to the cells, and then spin down the cells at 1,700 rpm at RT for 5 min.
- Discard the supernatant, wash the cells with 200 μL PBS, and spin down the cells at 1,700 rpm at RT for 5 min, then discard the supernatant.
- Resuspend the cells in 200 μL PBS and transfer the samples to FACs tubes for flow cytometric analysis.

*Note 1*: chemicals that block the secretion of proteins such as brefeldin A (BFA) are advised to be added during the last hours of incubation (4 – 6 hours before cell collection). BFA was not added in this study as the mode of action of the candidate adjuvants was yet to be fully understood.

## Supplementary Figures


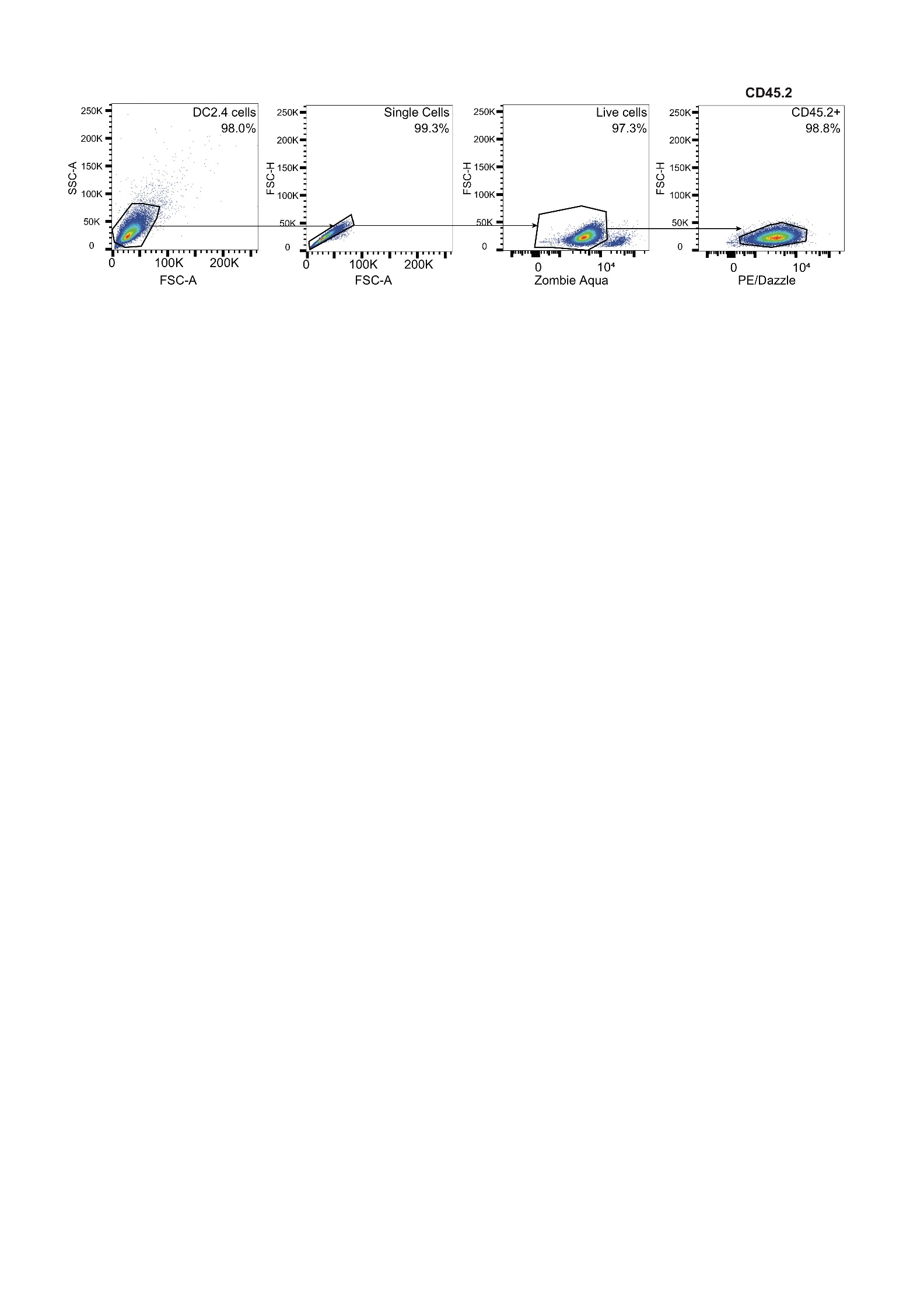


**Figure S1.** Gating strategies of DC2.4 cells. The cells were firstly gated based on forward scatter area (FSC-A, indicative of cell size) and side scatter area (SSC-A, indicative of cell granularity or complexity) profile (first plot). The second plot was gating on single cells to exclude doublets or clumps of cells, which was done by plotting FSC-A against FSC-H (forward scatter height) where single cells form a distinct population (second plot). The cells were then gated using Aqua live/dead staining to exclude dead cells (third plot). Finally, the cells were gated on CD45.2 (a marker expressed by C57BL/6 mice) positive populations (third plot).


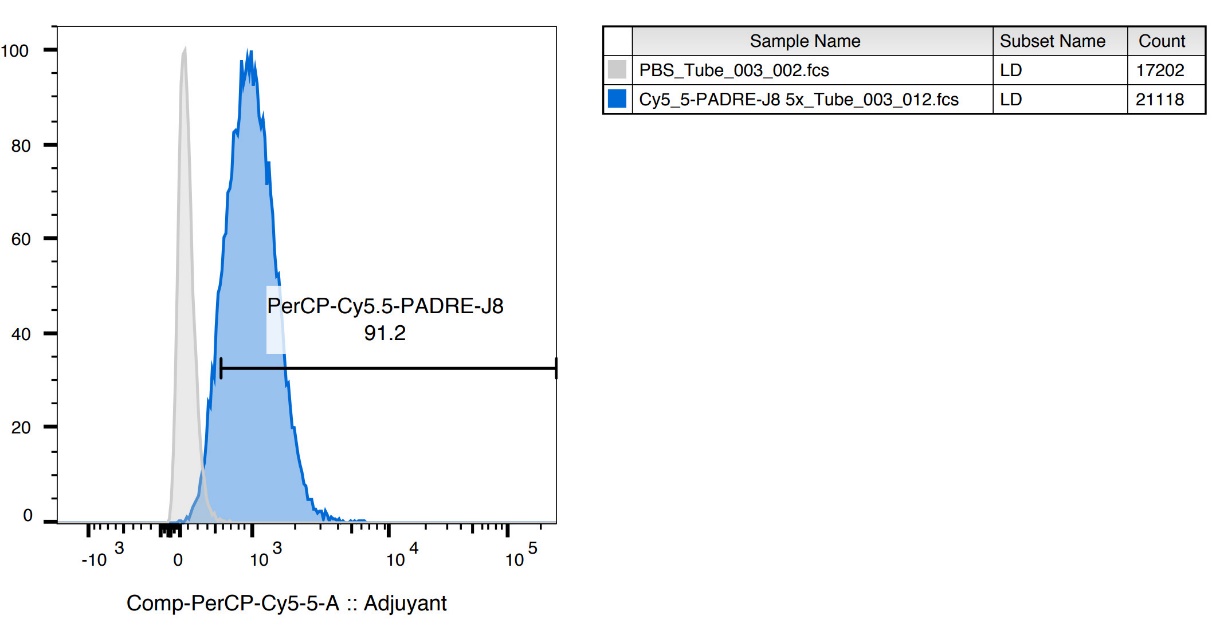


**Figure S2.** Uptake of Cy5.5-PADRE-J8 (1 μM) by DC2.4. Grey peak indicated the fluorescence intensity of PBS-treated cells, and blue peak indicated Cy5.5-PADRE-J8-treated (1 μM) cells. When the fluorescence-tagged antigen (e.g. Cy5.5-PADRE-J8) is co-delivered with adjuvants/delivery systems to enhance its uptake by DC2.4, titration of fluorescence-tagged antigen needs to be performed, as the signal of naked antigen without adjuvants or delivery systems here was saturated, and no difference would be observed if reporting percentage. In addition, reporting median fluorescence intensity (MFI) can be an alternative if signal by percentage of cells showing the fluorescence is saturated.


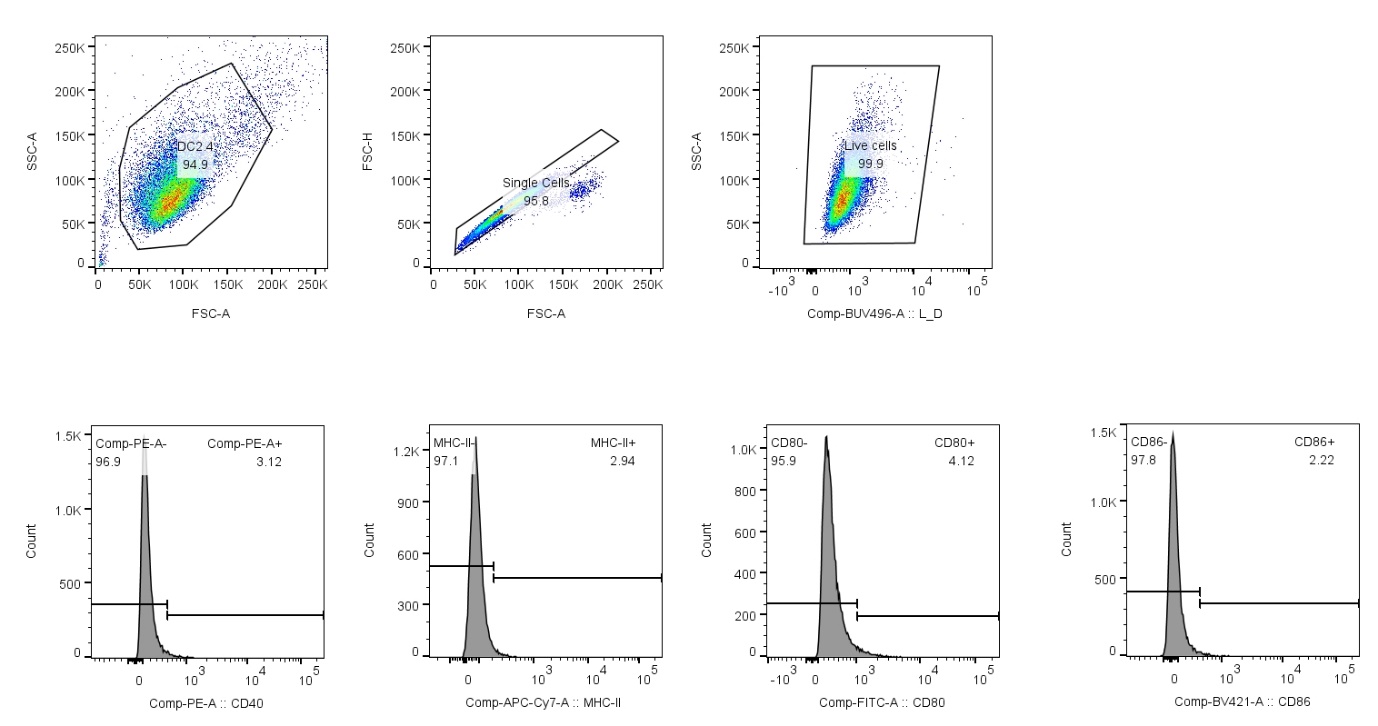


**Figure S3**. Gating strategies of DC2.4 cells for CD40, MHC-II, CD80 and CD86. The cells were firstly plotted by FSC-A against SSC-A (first plot), and single cell population was gated by plotting FSC-A against FSC-H (second plot), then live cell population was selected (third plot). The gating strategies to gate on live single cell populations were applied to all samples, then CD40 FMO, MHC-II FMO, CD80 FMO, or CD86 FMO samples were selected to present in histogram (x axis: CD40/MHC-II/CD80/CD86, y axis: histogram) to split the signal distribution into two open ended range gates for CD40, MHC-II, CD80, or CD86, respectively (fourth, fifth, sixth and seventh plots). Finally, the gating strategies were applied to all samples.


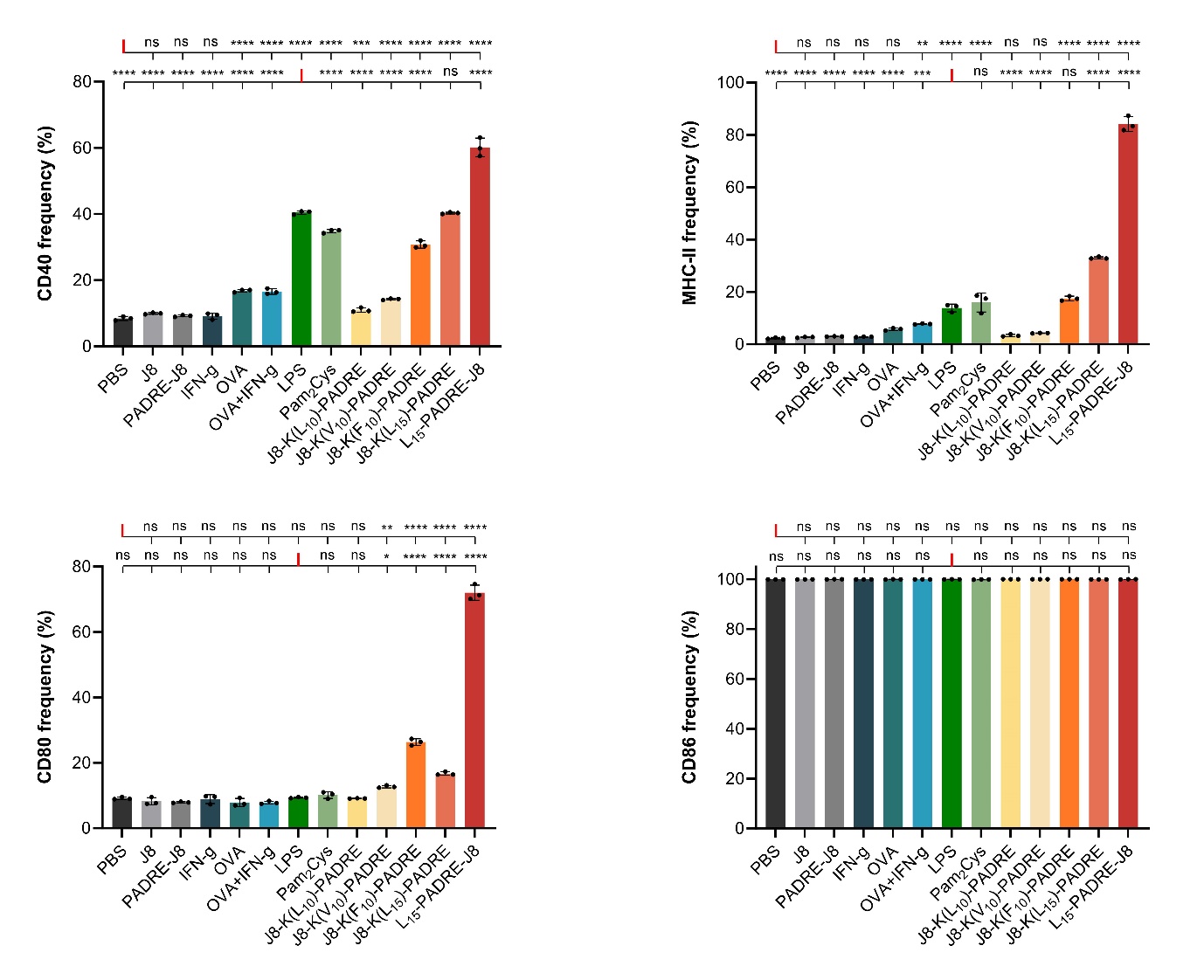


**Figure S4.** Frequency of cells expressing CD40 (top left), MHC-II (top right), CD80 (bottom left), and CD86 (bottom right). No significant difference was found between groups expressing CD86 post treatment with different compounds.


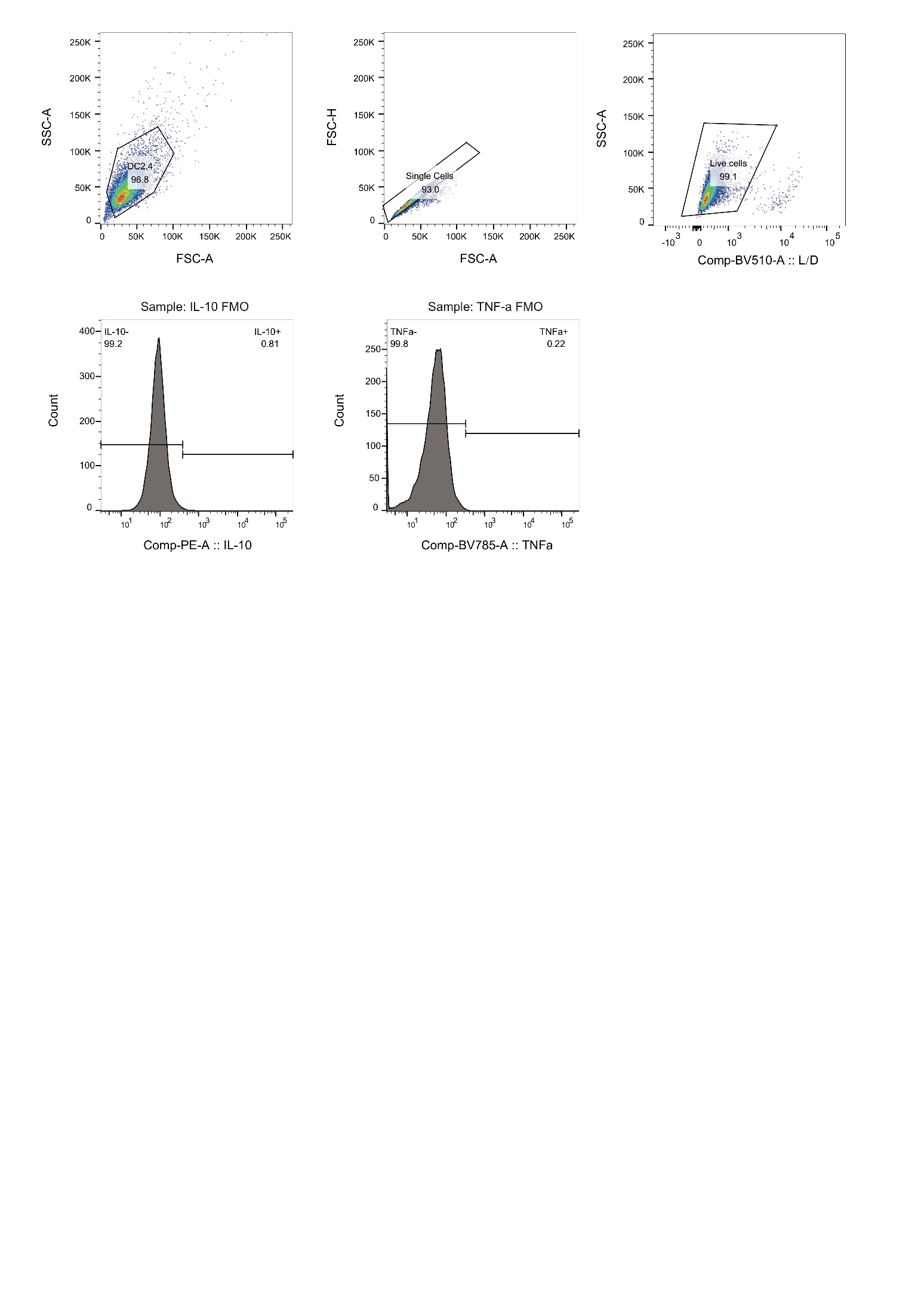


**Figure S5**. Gating strategies of DC2.4 cells for IL-10 and TNF-α production. The cells were firstly plotted by FSC-A against SSC-A (first plot), and single cell population was gated by plotting FSC-A against FSC-H (second plot), then live cell population was selected (third plot). The gating strategies to gate on live single cell populations were applied to all samples, then the IL-10 FMO and TNF-α FMO samples were selected to present in histogram (x axis: IL-10 or TNF-α, y axis: histogram) to split the signal distribution into two open ended range gates for IL-10 or TNF-α, respectively (fourth and fifth plots). Finally, the gating strategies were applied to all samples.

**
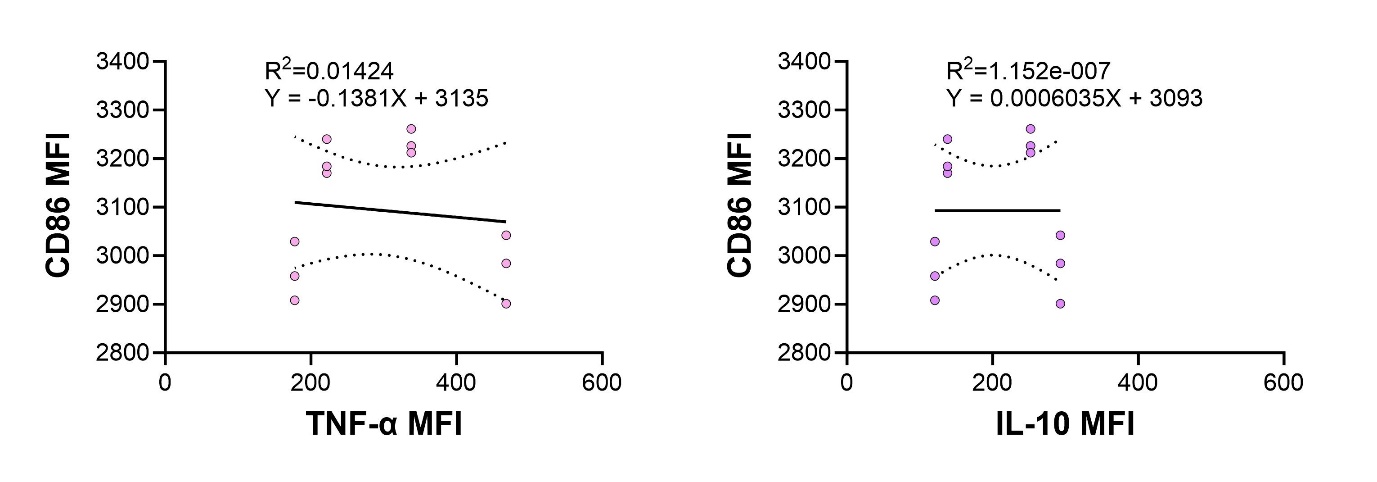
**

**Figure S6**. Correlation of CD86 MFI with TNF-α or IL-10 MFI in a linear regression model.

##
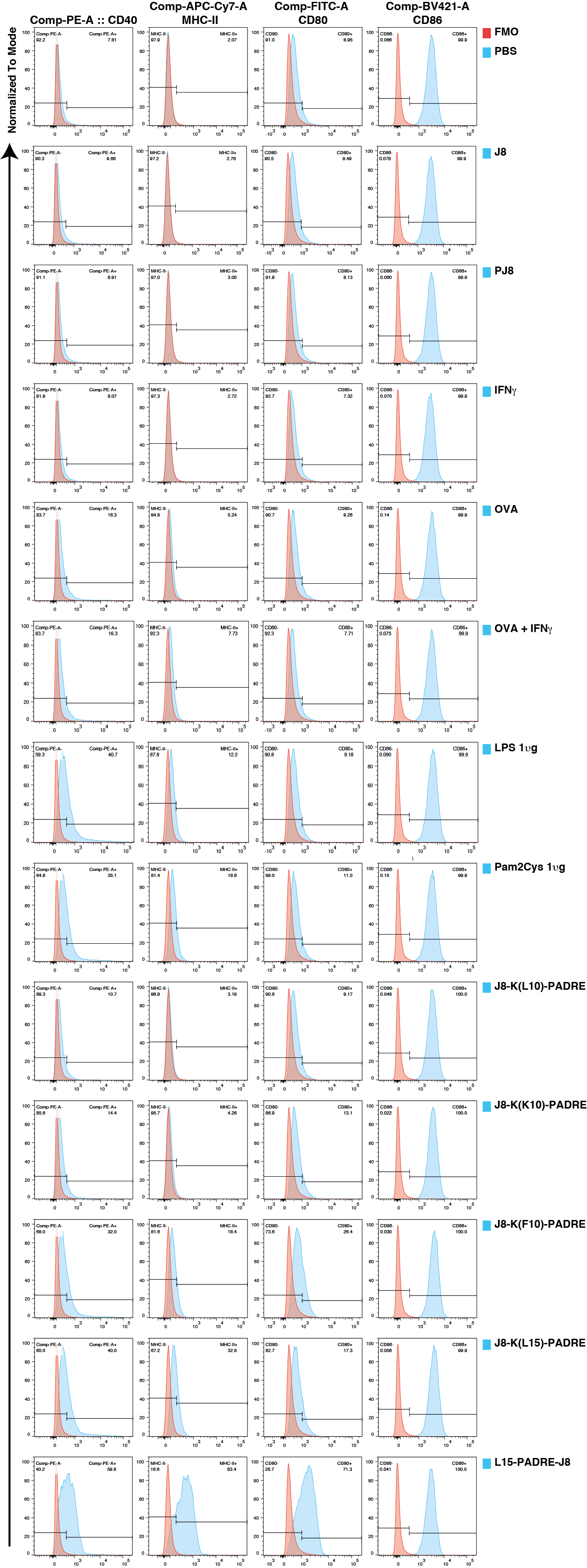


**Figure S7**. Individual histograms of treatment groups and surface markers. FMO= fluorescence minus one.

## References

[1] C. Dai, R.J. Stephenson, M. Skwarczynski, and I. Toth, Application of Fmoc-SPPS, Thiol-Maleimide Conjugation, and Copper(I)-Catalyzed Alkyne-Azide Cycloaddition “Click” Reaction in the Synthesis of a Complex Peptide-Based Vaccine Candidate Against Group A Streptococcus. in: W.M. Hussein, M. Skwarczynski, and I. Toth, (Eds.), Peptide Synthesis: Methods and Protocols, Springer US, New York, NY, 2020, pp. 13-27.
